# Thigh textiloma: a case report of a rare iatrogenic mass following orthopedic surgery

**DOI:** 10.1097/RC9.0000000000000217

**Published:** 2026-03-13

**Authors:** Rim Dhahri, Sarra Mahjoub, Khalil Amri, Lobna Ben Ammar, Moncef Aloui, Imen Gharsallah

**Affiliations:** a Department of Rheumatology, Military Hospital of Instruction of Tunis, Tunis, Tunisia; b Department of Orthopaedic, Military Hospital of Instruction of Tunis, Tunis, Tunisia; c Department of Radiology, Military Hospital of Instruction of Tunis, Tunis, Tunisia

**Keywords:** case report, gossypiboma, musculoskeletal foreign body, orthopedic surgery complication, textiloma

## Abstract

**Introduction and importance:**

Textiloma refers to a mass lesion caused by the iatrogenic retention of surgical gauze. Although well described in abdominal and thoracic surgery, its occurrence following musculoskeletal interventions remains rare and underreported.

**Case presentation:**

We report the case of a sexagenarian man presenting with swelling of the left thigh, associated with pain. His medical history was notable for a left femoral neck fracture treated 12 years earlier with locked intramedullary nailing. While hematological and biochemical tests showed no abnormalities, plain radiography and MRI suggested either chronic osteomyelitis with fistulization or a tumor, particularly a sarcoma. Given the diagnostic uncertainty, the therapeutic decision was to perform excision of the collection with debridement and lavage, followed by postoperative antibiotic therapy. The bacteriological analysis returned negative, while the pathological examination revealed a foreign body (gauze) that polarized under polarized light. These findings were consistent with a textiloma. The postoperative course was favorable, with good clinical and biological recovery and no subsequent complications.

**Clinical discussion:**

Textiloma is an iatrogenic retained surgical material that can trigger either an early exudative reaction or a delayed response mimicking tumors. Reported cases of thigh textilomas show variable latency periods and often present as masses with misleading imaging findings. Although rare, they may cause diagnostic confusion, recurrence, or even raise concerns about malignant transformation, highlighting the need for preventive measures and awareness.

**Conclusion:**

Textiloma should systematically be considered in the differential diagnosis of any postoperative mass. Increased awareness of this entity is essential to avoid misdiagnosis and prevent avoidable morbidity.

## Introduction and importance

Textiloma, also known as gossypiboma, muslinoma, gauzoma, or cottonoid, refers to a mass lesion caused by the iatrogenic retention of a foreign body within tissues^[^1,2^]^. As these terms are often used interchangeably in the literature, the term textiloma will be used throughout this manuscript for consistency.

While more commonly reported following abdominal or pelvic surgeries due to the larger potential spaces, textiloma is a rare complication in musculoskeletal surgery^[^3–7^]^.

It is an infrequently reported event due to surgeons’ reluctance, given the potential medicolegal issues they may face and the associated risks.

HIGHLIGHTS

- Textiloma is a rare iatrogenic complication that can be caused by retained surgical gauze after musculoskeletal surgery.
- It can mimic tumors or infections on imaging, making diagnosis challenging even years after surgery.
- Surgical excision with histopathological confirmation is essential for definitive diagnosis.
- Awareness and preventive measures are crucial to avoid this entirely preventable event.
- Consider textiloma in the differential diagnosis of masses at previous surgical sites to prevent unnecessary treatments.

Historically, the first reported case of a retained foreign body was described by Wilson^[^8^]^, who encouraged the documentation of similar cases. This initiative increased awareness and prompted surgical teams to implement preventive measures to reduce the risk of retained sponges. However, despite these efforts, it remains an ongoing problem, and the true incidence and prevalence of textiloma remain difficult to determine precisely^[^9^]^.

We report the case of a sexagenarian man presenting with swelling of the left thigh, associated with pain, whose diagnostic and therapeutic management led to the identification of a thigh textiloma.

This case report has been prepared in accordance with the SCARE checklist^[^10^]^.

## Presentation of case

A sexagenarian man presented to the outpatient consultations at the Rheumatology Department, with a swelling of the left thigh, associated with pain rated at 7/10 on the visual analog scale (VAS). His past medical history was notable for a left femoral neck fracture 12 years ago, which had been treated with locked intramedullary nailing. The postoperative course was complicated by the development of a nonunion 1 year later, for which he underwent corrective surgical treatment. Eventually, the osteosynthesis hardware was removed 2 years after the occurrence of the fracture.

Ten years after the last surgical procedure, the patient began to notice a slow-growing swelling accompanied by pain, without any skin changes. There was no history of trauma, systemic symptoms, weight loss, or fever. This led him to present to our department.

On admission, physical examination revealed that the patient had no fever and was able to walk unaided and without limping. There was a painful swelling in the middle third of the left thigh, associated with tenderness but without skin lesions. The adjacent joints, including the hip and knee, were free of pain and limitation.

The chronology of the patient’s orthopedic interventions and the development of clinical manifestations over time are outlined in Table 1.

**Table 1:** Timeline from initial surgery to diagnosis.

Time/years ago	Event/intervention	Observations
12 years before consultation	Left femoral neck fracture	Treated with locked intramedullary nailing
11 years before consultation	Complication: nonunion	Corrective surgical intervention
10 years before consultation	Removal of osteosynthesis hardware	No major complications reported
0–10 years after last surgery	Asymptomatic	-
10 years after last surgery	Onset of slow-growing swelling in the left thigh	Pain VAS 7/10
Current consultation	Presentation to Rheumatology Department	Painful mass in the middle third of the left thigh, joints free, no fever, no systemic signs, and no skin changes

The hematological tests showed no abnormalities, with a normal cell count and the biochemical tests were also normal.

**Figure 1.**
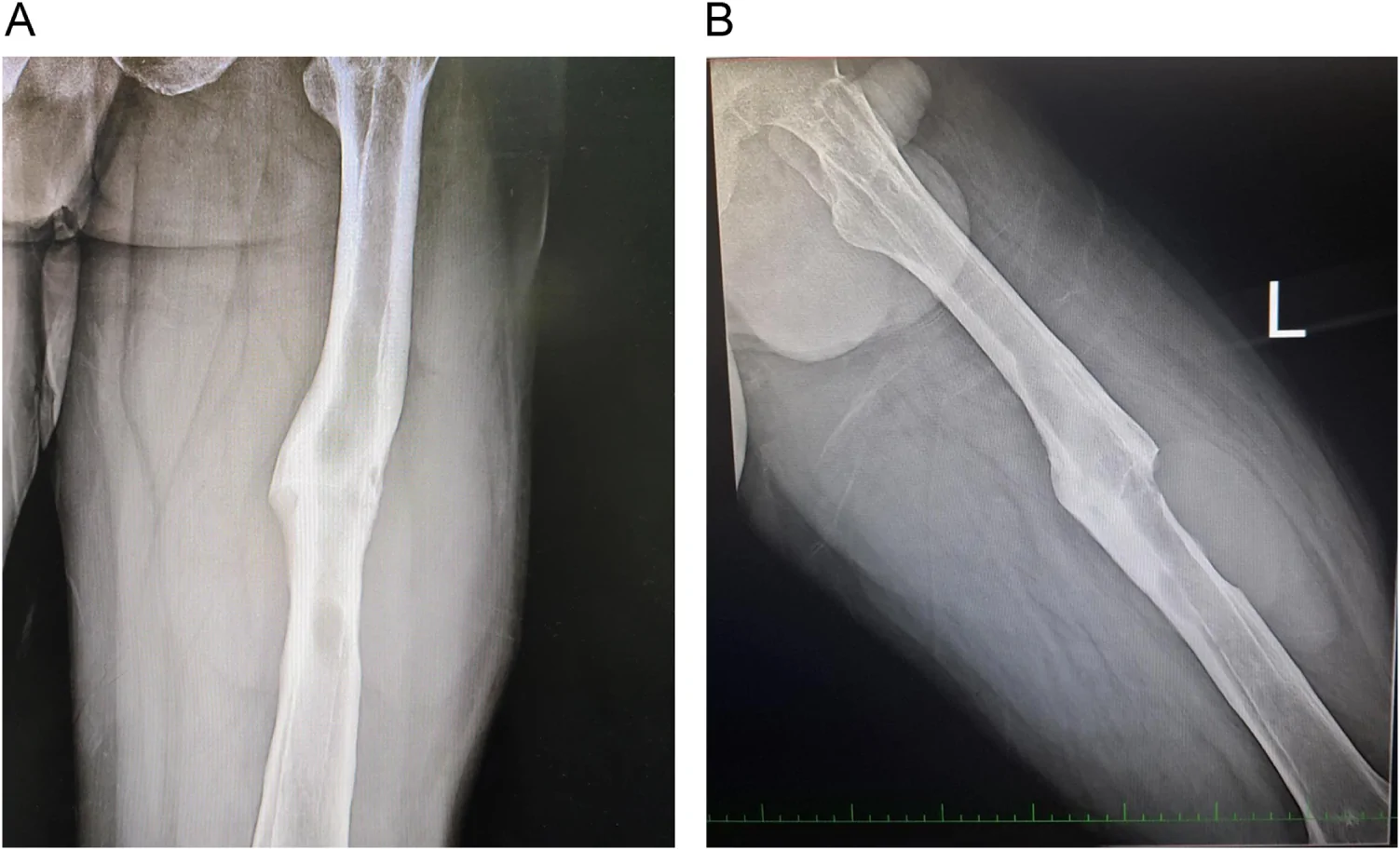
(A and B) Plain radiography of the left femur; anteroposterior view (Fig. 1A) and lateral view (Fig. 1B) show a healed mid-diaphyseal fracture of the femoral neck and osteolysis on the posterior surface.

Subsequently, a plain radiograph of the left femur revealed a healed mid-diaphyseal fracture of the femoral neck, with an area of osteolysis affecting the posterior surface of the femur (Fig. 1A and 1B).

**Figure 2.**
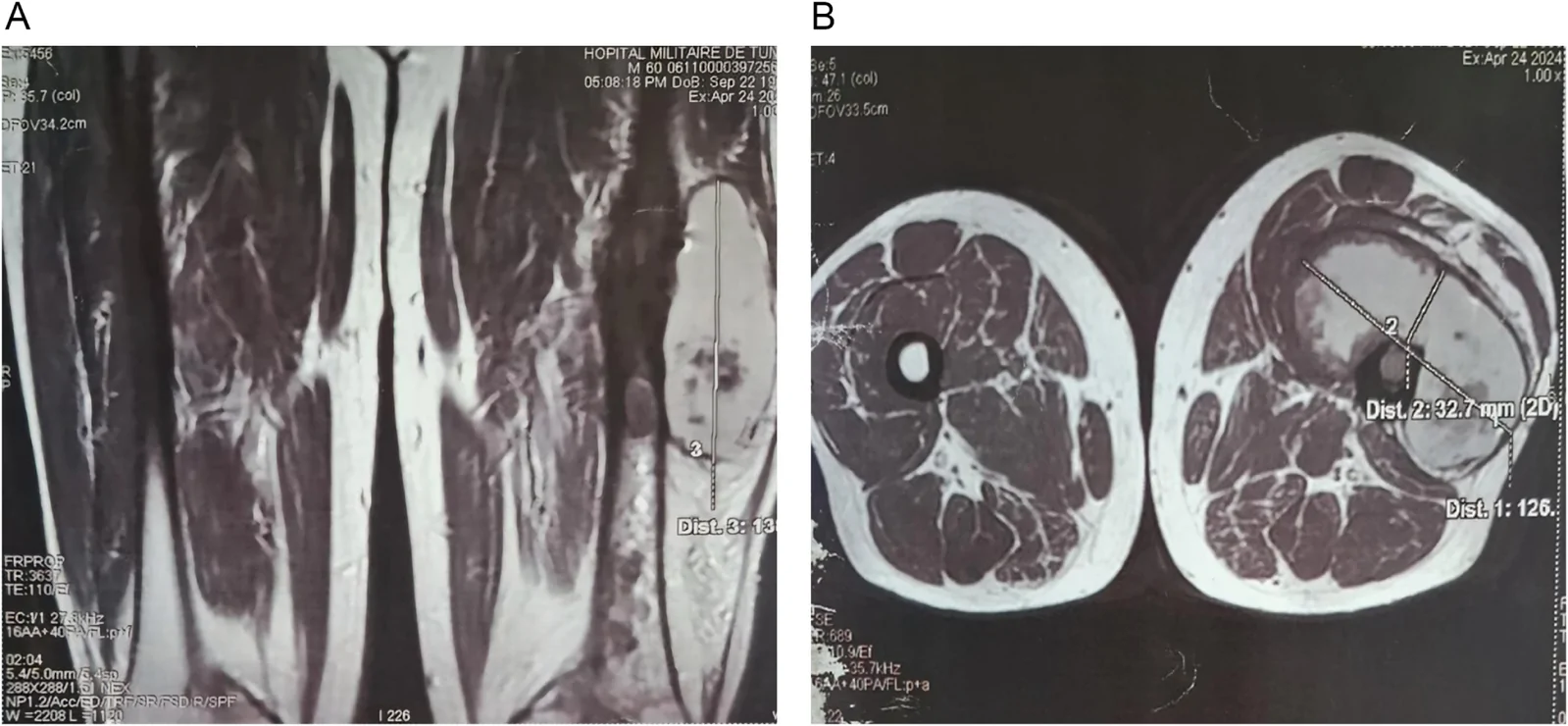
(A and B) Magnetic resonance imaging of the left thigh in sagittal (Fig. 2A) and axial views (Fig. 2B) showing the collection.

Magnetic resonance imaging (MRI) of the region revealed a deep collection in the left thigh located within the intermuscular aponeurosis, showing hyperintensity on T1, T1 Fat-Sat, and isointensity on T2. It was septated with fine partitions, hypointense on T1 and T2, and did not enhance after gadolinium injection. The collection was heterogeneous, with hypointense areas surrounded by a thickened, moderately enhancing peripheral shell. It measured 126 mm by 33 mm transversely and 139 mm in height. This collection communicated with the bone marrow of the left femur. Additionally, there was a second subperiosteal collection in the middle third of the left femur, measuring 15 mm by 9 mm transversely and 29 mm in height, with surrounding hyperintensity on both T1 and T2. (Fig. 2A and 2B). While the orthopedic surgeons considered either chronic osteomyelitis with fistulization or a tumor, particularly a sarcoma, the reporting radiologist suggested a differential diagnosis of a liquefied hematoma.

The decision was then made to proceed with surgical biopsy. A longitudinal incision was performed using an external crural approach, centered over the area of maximal swelling. Upon opening the deep fascia, pressurized fluid was released.

Surgical exploration revealed fibrous material consistent with a retained surgical sponge. A large encapsulated mass was found, adherent to the anterolateral surface of the femur, containing necrotic and inflammatory tissue.

Intraoperatively, the appearance was consistent with a textiloma, measuring approximately 100 × 50 mm. Complete excision of the mass was carried out, and fibrous extensions into the surrounding tissues were removed when possible.

**Figure 3.**
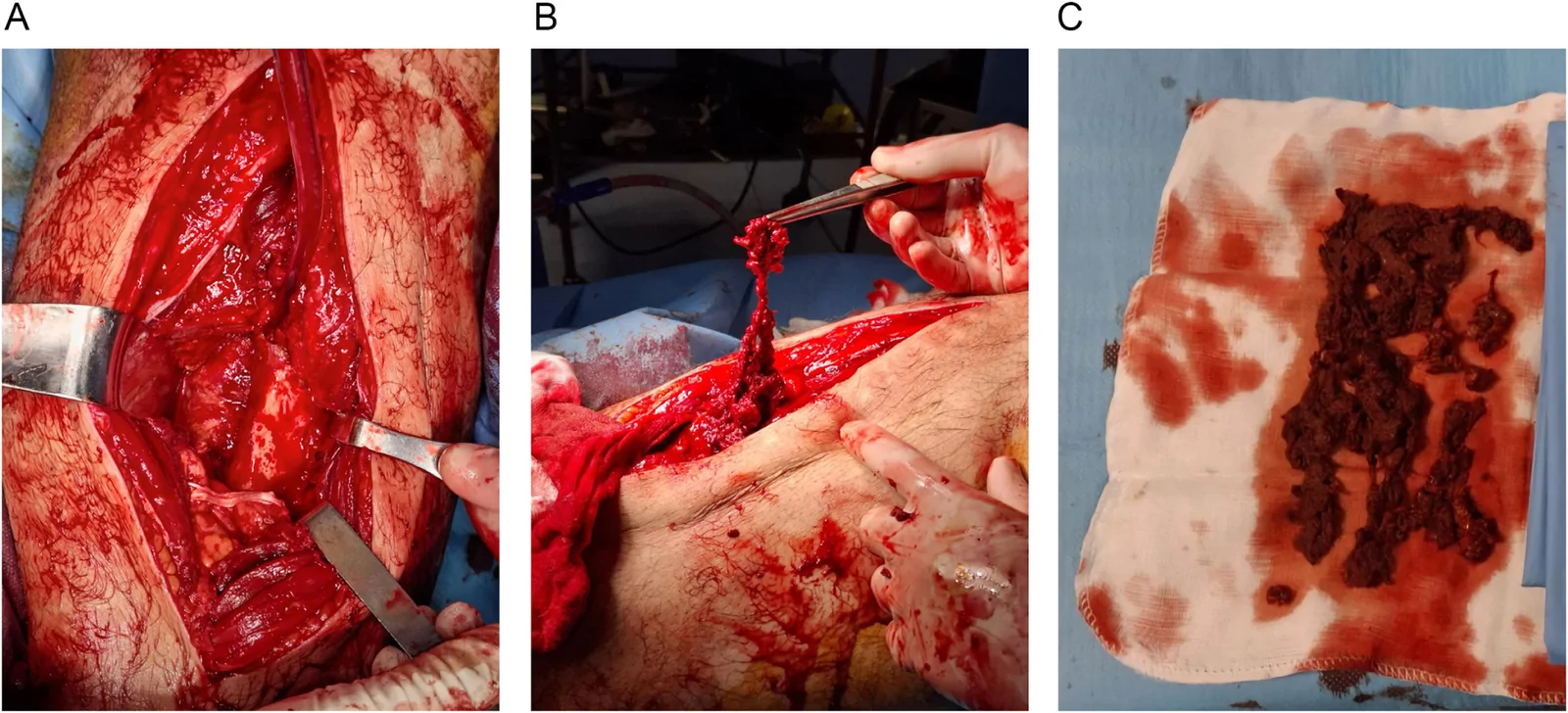
(A–C) Intraoperative views of the textiloma before extraction (Fig. 3A), during extraction (Fig. 3B), and after extraction (Fig. 3C).

The patient underwent excision of the collection, debridement, and lavage, followed by postoperative antibiotic therapy (Fig. 3A–3C).

Two samples were taken peroperatively and before any initiation of antibiotic therapy: one for bacteriological analysis, which was negative, and one for pathological examination, which revealed fibrinous and blood-derived material containing numerous vegetable fibers, corresponding to foreign bodies (gauze) that polarize under polarized light.

These findings were consistent with a textiloma measuring 100 × 100 mm, surrounded by a capsule measuring 60 × 50 × 53 mm, composed of inflammatory fibro-adipose tissue without specific features or signs of malignancy.

Up to 3 months postoperatively, the patient experienced complete regression of symptoms, including pain and swelling, with good consolidation at the surgical site.

## Clinical discussion

This is a case of a sexagenarian man with painful swelling of the left thigh, 12 years after a left femoral neck fracture treated with intramedullary nailing. Imaging suggested chronic osteomyelitis or a soft tissue tumor, particularly a sarcoma. Surgical excision with debridement was performed; bacteriology was negative, and histopathology revealed retained surgical gauze consistent with a textiloma.

This case highlights the diagnostic challenge posed by textilomas and serves as an example of how retained surgical materials can present long after the initial procedure.

In fact, any surgical procedure may require materials like cotton pads, gauze, and sponges to achieve hemostasis, making the risk of accidental retention a possibility that may lead to various clinical and paraclinical manifestations.

When exposed to a foreign body, the human body can exhibit two types of responses^[^11^]^. The first is an exudative reaction that results in abscess formation, which may cause fluid accumulation or evolve into a fistula or draining sinus. These cases typically present early. The second is less common and consists in a sterile fibrinous response, leading to the formation of a foreign-body granuloma, often encapsulated by a thick fibrous layer, which usually confuses the diagnosis, leading to a suspicion of neoplasm.

It is worth noting that the clinical presentation of textilomas is non-specific and that the interval between surgery and symptom onset varies. It has been reported to extend up to 40 years, making preoperative diagnosis particularly challenging in some cases^[^5,12^]^.

We have collected other reported cases of thigh-located textilomas and analyzed their specific characteristics, along with the circumstances of occurrence^[^3,6,13^]^. Table 2 summarizes details on age, sex, previous surgery, interval to diagnosis, textiloma location, symptoms, and outcomes of these cases.

**Table 2:** Summary of reported cases of textilomas.

Reference	Age/sex	Previous surgery	Interval	Location of textiloma	Symptoms/outcome
D.F. Kalbermatten (2001)	41/man	Internal fixation of a femoral fracture	20 years	Right thigh	Swelling and weakness of the left thigh/healing
A. Puri (2006)	58/female	Internal fixation of an intertrochanteric fracture	13 years	Proximal part of the medial aspect of the right thigh	Pain and difficulty in walking/recurrence of textiloma in 6 years
B. MBOTI (2001)	59/man	Internal fixation of a proximal femur fracture	23 years	The proximal left femur	Pain, tenderness, and loss of function revealing a pathological fracture/healing
K. Sakayama (2004)	61/ man	External skeletal fixation of an open fracture	40 years	Left thigh	Gradually enlarging swelling/uneventful
F.W. Abdul-Karim (1992)	50/woman	Internal fixation of a comminuted femoral fracture	35 years	Left thigh	Pain and a progressively enlarging fullness

Concerning the imaging characteristic features, textilomas usually present with a whirl-like pattern on radiographs and CT scans, resulting from gas produced by bacteria trapped in the gauze fibers, giving the mass a spongy appearance^[^14^]^.

This imaging feature of textilomas is relatively uncommon and is most frequently reported in digestive and gynecological surgeries. While in orthopedic surgery, particularly in limb procedures, it is less frequently observed, making the diagnosis even more challenging on imaging.

It is well known that MRI is highly useful in the localization of non-metallic foreign bodies in soft tissues, as well as in lesion staging and identifying the nature of lesions in problematic cases^[^15^]^. Additionally, it has been reported to reveal the folded fabric structure of the gauze on T2-weighted images in some cases^[^16^]^.

Scintigraphy, however, has been noted for its high negative predictive value, which can be useful in ruling out malignancy^[^5,17,18^]^.

Table 3 summarizes the various radiological features of other reported cases of thigh-located textilomas, as observed on standard radiographs and MRI, along with the final histopathological outcomes.

**Table 3:** Radiological features and histopathological outcomes of thigh-located textilomas.

Reference	Location of textiloma	Plain radiography	MRI	Anapath
D.F. Kalbermatten (2001)	Right thigh	A massive osteolytic destructive process surrounded by a radio-opaque rim	A large, sharply marginated tumor with an inhomogeneous signal	Organizing granulation tissue mixed with amorphous fibrin
A. Puri (2006)	Right thigh	A large soft-tissue mass with evidence of bony remodeling	A thigh lesion predominantly involving the adductor muscles, hypo- to isointense on T1-weighted images, heterogeneously hyperintense on T2-weighted images with ill-defined nodular hypointense areas, measuring 180 × 155 × 140 mm	Coagulative necrotic material, fibrocollagenous tissue, and chronic inflammatory infiltrate
B. Mboti (2001)	Left femur	A complex fracture arising in a lytic tumor with smooth scalloped borders	A bone defect with heterogeneous lesions encircled by gadolinium-enhancing areas, and a mass situated on the posterolateral aspect of the femur	Atypical proliferation of bone without conclusive diagnosis
K. Sakayama (2004)	Left thigh	Soft tissue mass with osteolysis and periosteal thickening	A well-marginated soft tissue mass isointense on T1 and heterogeneous signal intensity on T2 with a non-homogeneous contrast enhancement in the periphery	A fibrous foreign body with reactive changes
F.W. Abdul-Karim (1992)	Left thigh	Homogeneous soft-tissue density mass without calcification and a well-defined erosion bordered by a sclerotic rim of the femoral diaphysis and associated to bone remodeling	An eccentric, nonhomogeneous, soft-tissue mass of low signal on T1 and an admixture of high and low signal on T2-weighted images. The cortex of the femur was thinned with no evidence of cortical invasion	A fibrous, walled hematoma with organization, fibrosis, and a prominent foreign body giant cell and histiocytic reaction with polarizable foreign material of a surgical sponge

If left undiagnosed, retained surgical gauze may undergo progressive degradation over time and become surrounded by a fibrous capsule. Some authors have speculated that this chronic inflammatory environment, containing mesenchymal cells, could theoretically contribute to sarcoma development^[^19,20^]^.

However, the association between retained foreign bodies and sarcoma remains highly controversial and is based exclusively on isolated case reports and small series, rather than on robust epidemiological evidence. A published review reported a limited number of sarcomas, including six angiosarcomas and 40 other sarcoma subtypes, occurring in association with various foreign materials, as well as two angiosarcomas specifically linked to surgical sponges^[^19,20^]^.

However, the association between retained foreign bodies and sarcoma remains highly controversial and is based exclusively on isolated case reports and small series, rather than on robust epidemiological evidence. A published review reported a limited number of sarcomas, including six angiosarcomas and 40 other sarcoma subtypes, occurring in association with various foreign materials, as well as two angiosarcomas specifically linked to surgical sponges.

Although extremely rare, recurrence of a textiloma after excision is still possible, as previously reported^[^13^]^. This can lead to confusion, especially with neoplasms, and may even result in unnecessary, exhaustive therapeutic approaches that could have been entirely avoided.

Our case highlights the importance of preventing such incidents, which are solely the responsibility of the operating surgeon. For this reason, the linen count method was introduced, along with the use of sponges with radiopaque markers, which aid in locating sponges in the event of discrepancies in the linen count.

However, this method has not been successful in eliminating the incident^[^21^]^, as the use of sponges with radiopaque markers is still not a widespread practice in the developing world, particularly in Tunisia.

Since these lesions are often confused with neoplasms, they can cause significant anxiety for the patient, particularly regarding the potential for mutilating surgery. In the absence of histopathological evidence confirming the malignant nature of the lesion, it is prudent to consider a textiloma diagnosis, especially at the site of a prior surgery, regardless of how much time has passed since the incident.

This approach allows for a more conservative surgical technique rather than a radical resection.

While awaiting future advancements, it is essential not to exclude textilomas from the differential diagnosis of an incidental mass in a patient with a previous surgical history, just as one must also consider the possibility of malignant tumors.

## Conclusion

This case underscores the importance of maintaining a high index of suspicion for textiloma when evaluating a mass at a previous surgical site. Considering this diagnosis early may allow for a less morbid surgical approach and prevent unnecessary patient distress and overtreatment. Given that textiloma is a purely iatrogenic and entirely preventable complication, continuous reinforcement of preventive measures, such as strict sponge-counting protocols and the use of radiopaque markers, remains essential.

## Data Availability

The data that support the findings of this study are available from the corresponding author upon reasonable request.
